# Prognostic factors for wound complications after childbirth‐related perineal trauma: A systematic review and meta‐analysis

**DOI:** 10.1111/aogs.70041

**Published:** 2025-08-20

**Authors:** Rebecca Man, Tanvi Bhatia, Alice Sitch, R. Katie Morris, V. Hodgetts Morton, the CHAPTER group, Laura Jones, Laura Magill, Christine MacArthur, Sara Webb, John Maltby, Sarah Hillman, Nicola J. Adderley, Olalekan Lee Aiyegbusi, Marian Knight, Krishnarajah Nirantharakumar

**Affiliations:** ^1^ Department of Applied Health Sciences, School of Health Sciences, College of Medicine and Health University of Birmingham Birmingham UK; ^2^ Warwick Hospital South Warwickshire University NHS Foundation Trust Warwick UK; ^3^ National Institute for Health and Care Research (NIHR) Birmingham Biomedical Research Centre Birmingham UK; ^4^ Birmingham Women's Hospital Birmingham Women's and Children's NHS Foundation Trust Birmingham UK

**Keywords:** postnatal, postpartum, risk factor, wound infection

## Abstract

**Introduction:**

Although childbirth‐related perineal trauma affects the majority of women after vaginal birth, very few healthcare resources are allocated to reducing morbidity from perineal trauma. Wound complications are frequent after perineal trauma has been sustained; however, we know little about which factors are predictive of developing a wound issue. To target possible interventions effectively, it is crucial that those at higher risk are identified. Here, we perform a systematic review and meta‐analysis of prognostic factors for sustaining wound complications after childbirth‐related perineal trauma.

**Material and Methods:**

Medline, Embase, Web of Science, and CINAHL were searched from inception to December 2024 using relevant search terms. There were no restrictions on language or year of publication. Observational studies that investigated two or more potential prognostic factors for wound complications after childbirth related‐perineal trauma, where adjusted risks were calculated, were eligible for inclusion. We included all types of tears, sustained through spontaneous or assisted vaginal birth. Meta‐analysis was performed where five or more studies investigated a particular prognostic factor for perineal wound complications. Odds ratios (ORs) were pooled using a random effects model. The review was prospectively registered in PROSPERO (CRD42023458738).

**Results:**

Fifteen studies were eligible for inclusion, involving 71409 women. Studies included were published between 2006 and 2024 across six different countries. Assisted vaginal birth (10 studies, 65 375 women: OR 2.77, 95% confidence interval [CI] 1.89–4.06) was a significant risk factor for wound complication. Raised body mass index (six studies, 64 770 women: OR 1.33, 95% CI 0.56–3.18) was not a significant risk factor. Prolonged second stage of labor, smoking, and episiotomy were each investigated in three primary studies; therefore, data was insufficient for meta‐analysis; however, individual studies indicated that there might be an association with perineal wound complication.

**Conclusions:**

Assisted vaginal birth is a significant risk factor for perineal wound complication after childbirth‐related perineal trauma. Overall, there are limited studies investigating prognostic factors for perineal wound complication after childbirth related‐perineal trauma. Whilst we highlight potential prognostic factors, we recommend that a robust, well‐powered primary research study with clearly defined wound complication outcomes and prognostic factors is needed.

## Graphical abstract

Assisted vaginal birth is a risk factor for perineal wound complication. This review was limited by a paucity of existing research in the area; therefore, further robust and well‐powered primary research is required. Created in BioRender. Man, R. (2025) https://BioRender.com/3zsekbj.

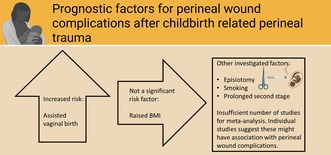

**Abbreviations**
aOR: adjusted odds ratio
aRR: adjusted risk ratio
BMI: body mass index
CI: confidence interval
OR: odds ratio

**Key message**

Assisted vaginal birth is a significant risk factor for perineal wound complications in the postpartum period after meta‐analysis. There is a paucity of research investigating prognostic factors for perineal wound complications after childbirth related‐perineal trauma. Therefore, further robust and well‐powered primary research is required.

## 1 INTRODUCTION

There is a substantial and urgent need for improvements to and research around postnatal care—the recent Birth Trauma governmental UK report highlighted poor postnatal care as an almost universal theme.^1^ The report emphasized the potentially devastating impact of birth injuries, particularly surrounding perineal trauma and the lasting consequences of perineal wound infections.^1^ Perineal trauma affects the vast majority of women after vaginal childbirth.^2^ Although childbirth‐related perineal trauma is the most common complication after childbirth, and problems with subsequent wound healing can have detrimental effects on both physical and psychological health, little is known about which factors may predispose to postpartum wound issues.^3^, ^4^, ^5^, ^6^

Estimates for wound infection after childbirth related‐perineal trauma vary widely with population, tear type, and differing definitions of infection, ranging between 0.1 and 23.6%.^7^ Currently in the United Kingdom, women who sustain the most severe types of tear—third‐ and fourth‐degree tears—receive prophylactic antibiotics, in addition to planned follow‐up with a specialist.^8^ Women who experience first/second‐degree tears or episiotomies after spontaneous vaginal birth do not routinely receive any postnatal interventions or targeted care to reduce wound‐related morbidity.

As healthcare resources are finite and interventions often not without unintended consequences, knowledge of which factors convey a greater risk for postpartum wound morbidity may help to target any future interventions more effectively. By intervening early and using targeted prophylactic measures in those at risk, increasingly serious complications may be avoided. To our knowledge, there is no existing systematic review which synthesizes existing studies investigating prognostic factors for wound complications after childbirth related‐perineal trauma. Through this systematic review and meta‐analysis, we investigate prognostic factors for wound complications after childbirth related‐perineal trauma. This study forms part of the wider CHAPTER (Childbirth Acquired Perineal Trauma) program, aiming to improve care for women who have sustained perineal trauma.^9^

## 2 MATERIAL AND METHODS

### 2.1 Study design and registration

This is a systematic review and meta‐analysis of observational studies which evaluate potential prognostic factors for wound complications after childbirth‐related perineal trauma. The review was completed following the Preferred Reporting Items for Systematic Reviews and Meta‐Analyses (PRISMA) guidance; the systematic review protocol was published prospectively through the overarching PROSPERO (CRD42023458738).^10^, ^11^

### 2.2 Inclusion and exclusion criteria

Observational studies including two or more potential prognostic factors for perineal wound complication were included where odds ratios (ORs) were available. We included only studies that had provided adjusted estimates for the purposes of this review, as the aim was to identify prognostic factors that persisted independently, to ensure the findings have direct relevance to a clinical setting. Inclusion of both adjusted and unadjusted results in the same analysis would not allow for meaningful conclusions to be drawn. The inclusion of unadjusted results in separate analyses would not confidently allow those more at risk of a wound complication to be identified in a clinical setting, as the prognostic factor may not persist independently. All degrees of perineal tear sustained through spontaneous or assisted vaginal birth were included. Non‐English studies were translated. Studies investigating prognostic factors for composite measures of postpartum infection (e.g., including endometritis, mastitis and urinary tract infection in addition to perineal wound complication) were excluded if the prognostic factors for perineal wound complication were not available separately. Studies investigating only prognostic factors for sustaining severe perineal trauma (e.g., third‐ and fourth‐degree tears), as opposed to prognostic factors for perineal wound complication, were excluded, as this was not aligned with our research question.

### 2.3 Outcomes and prognostic factors

All potential prognostic factors with adjusted estimates provided by study authors for perineal wound complications were recorded in our systematic review. Where different perineal wound complications were investigated by study authors (e.g., wound dehiscence/breakdown, wound infection, composite wound complication) and adjusted odds ratios (aORs) were provided separately for prognostic factors for each outcome, all prognostic factor estimates for each outcome provided by authors were recorded during data extraction. Where the prognostic factor was amenable to meta‐analysis, if the authors provided the OR for multiple wound‐related outcomes, then the prognostic factor ORs for perineal wound infection were used preferentially for the purposes of meta‐analysis to avoid double counting. Perineal wound complications and prognostic factors were as per the definitions of primary study authors— definitions used in primary studies for both wound complications and prognostic factors were extracted and recorded in our systematic review.

### 2.4 Search strategy

Systematic literature searches were undertaken in Medline, Embase, CINAHL, and Web of Science. Searches were undertaken from database inception until December 2024 using a combination of medical subject headings and text words. Example search terms can be found in Appendix S1, with the search terms then adapted for each database.

### 2.5 Study selection and data extraction

Two reviewers (RM, VHM) independently reviewed titles and abstracts of the citations retrieved, with discrepancies resolved by consensus. The process was then repeated by the same authors for the studies proceeding to full‐text evaluation. Data extraction was completed by two reviewers independently (RM, TB), with any discrepancies resolved by consensus. Covidence software was utilized for the organization of citations and study screening. Where studies met the inclusion criteria, a pre‐designed electronic spreadsheet was used for data extraction. Authors of primary studies were contacted for more information where indicated (for example authors were contacted where it appeared there may be additional adjusted results not included in the primary publication or where it was not clear which variables had been adjusted for).

### 2.6 Risk of bias assessment

The Quality in Prognostic Factor Studies (QUIPS) tool was used to determine risk of bias for each included study.^12^ Two review authors conducted risk of bias assessments independently (RM, TB) with any discrepancies resolved by consensus. Risk of bias was determined for each of the six domains using the signaling items and descriptors specified in the QUIPS tool, with ratings deemed as high, moderate, or low.^12^, ^13^

### 2.7 Data analysis

Meta‐analysis was undertaken where five or more studies provided aOR data on the same prognostic factor for perineal wound complication. Data was synthesized using R and RStudio software using the meta package. Odds ratios log‐transformed and pooled using a random effects meta‐analysis to give an overall estimate and 95% confidence interval (CI) were calculated. Heterogeneity was measured using the Tau^2^ statistic to assess between‐study heterogeneity in effect sizes and 95% prediction intervals to ascertain the degree of effect size variation across studies. Where the 95% prediction interval crosses one (null effect) this demonstrates that there is heterogeneity in the effect size across populations and that in certain groups, null effect may be experienced or an effect in the opposite direction.^14^

### 2.8 Sensitivity and subgroup analyses

Sensitivity analyses were undertaken for prognostic factors where meta‐analysis had been performed, where primary study authors provided additional nonpublished results from multivariable logistic regression, e.g., where the results of each stage of backward stepwise elimination were provided. Where there were prognostic factors in earlier steps of the model, which were later eliminated and therefore not included in the final published model, the latest step at which the prognostic factor estimate appeared was included in sensitivity analyses only. Further post‐hoc sensitivity analyses were performed, first to exclude the results of any studies that may have been deemed eligible to include but that involved a small minority of women with more broad complications related to perineal trauma, in addition to wound‐related complications only. Additionally, we performed a post‐hoc sensitivity analysis removing results of studies that had not clearly adjusted for perineal trauma type. Subgroup analyses by type of perineal trauma were performed where there were ten or more studies included in the meta‐analysis.

## 3 RESULTS

**FIGURE 1:**
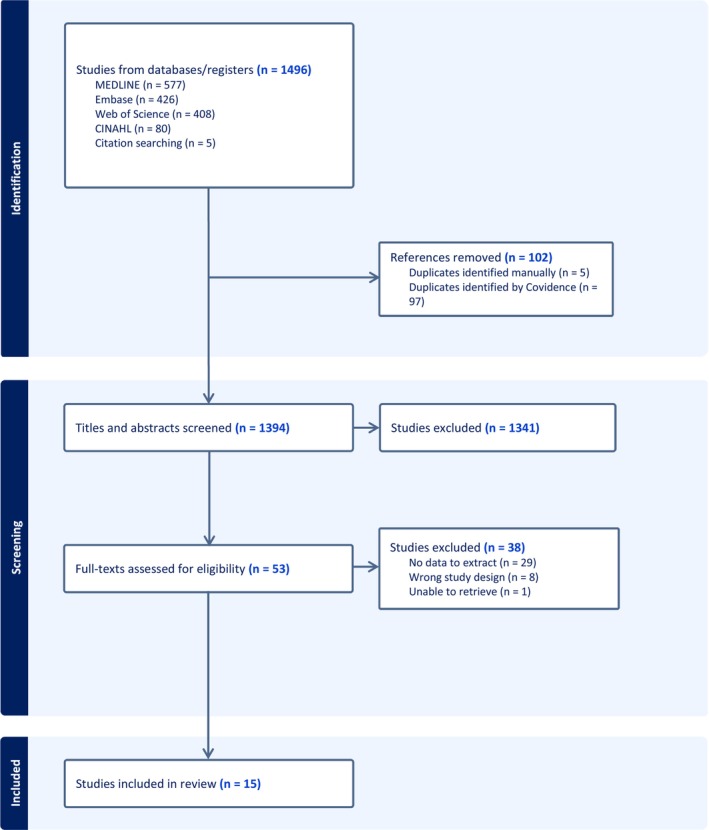
PRISMA flow diagram.

Fifteen studies were eligible for inclusion, involving 71 409 women (PRISMA flow diagram Figure 1).^15^, ^16^, ^17^, ^18^, ^19^, ^20^, ^21^, ^22^, ^23^, ^24^, ^25^, ^26^, ^27^, ^28^, ^29^ Studies were conducted across the United States (eight studies),^17^, ^19^, ^21^, ^23^, ^24^, ^26^, ^28^, ^29^ China (two studies), France (two studies),^20^, ^27^ Denmark (one study),^16^ India (one study),^18^ and Thailand (one study).^22^ We included seven retrospective cohort studies,^18^, ^21^, ^23^, ^25^, ^26^, ^27^, ^28^ four case‐controlled studies,^15^, ^17^, ^20^, ^24^ two prospective cohorts,^16^, ^19^ one retrospective cross‐sectional study,^22^ and one cohort study with retrospective and prospective components.^29^

Six studies included third‐ and fourth‐degree tears only^19^, ^21^, ^26^, ^27^, ^28^, ^29^; four studies included second degree or more severe tears^16^, ^17^, ^20^, ^24^; two studies investigated all tear types^22^, ^23^; one study included unspecified tear types^18^; one study included episiotomy only^25^; and one study included episiotomy and second‐degree tears.^15^ Thirteen studies included women who underwent spontaneous or assisted vaginal birth^15^, ^16^, ^17^, ^18^, ^19^, ^20^, ^21^, ^22^, ^24^, ^26^, ^27^, ^28^, ^29^; one study included women who underwent assisted vaginal birth only^23^ and one study included women who underwent spontaneous vaginal birth^25^ (unclear whether this study also included those who underwent assisted vaginal birth). Eight studies investigated prognostic factors for wound breakdown/dehiscence^15^, ^16^, ^17^, ^18^, ^19^, ^20^, ^23^, ^24^; six investigated a composite outcome including any wound complication^19^, ^21^, ^26^, ^27^, ^28^, ^29^ and four investigated perineal wound infection^16^, ^19^, ^22^, ^25^ (two studies investigated prognostic factors for more than one wound outcome^16^, ^19^). Characteristics of included studies, including individual study definitions of investigated prognostic factors and wound outcomes, can be found in Table 1.

**TABLE 1:** Study characteristics table.

First author	Year of publication	Country	Objective	Study design	Years in which patients recruited	Number included in study and number affected by wound complication	Mode/s of birth included	Type of perineal trauma	Complications for which risk factors were assessed including definition	Prognostic factors assessed and definition	Factors which were adjusted for in adjusted results^a^
Cui	2022	Hong Kong	To identify risk factors associated with the breakdown of perineal lacerations	Retrospective case–control	2018–2019	118 included in case–control study 59 women with perineal wound breakdown (0.6% from the 9224 women screened with a vaginal birth [infer denominator includes women with an intact perineum])	Spontaneous or assisted vaginal birth	Episiotomy and 2nd degree	1. Wound breakdown: superficial or full thickness separation of the skin or mucosa, with or without involvement of the anal sphincter and/or anal mucosa	1. Postpartum hemorrhage >500 mL, 2. Previous vaginal birth, 3. Assisted vaginal birth, 4. Working years for birth provider, 5. Meconium‐stained liquor, 6. Induction of labor, 7. Labor augmentation	Antibiotics in postpartum labour, estimated blood loss >500 mL, assisted vaginal birth, meconium stained liquor, induction of labour, labour augmentation, working years for birth provider
Freret	2023	USA	To describe the rate of antibiotic administration for obstetric anal sphincter injuries, to characterize factors associated with antibiotic administration on the day of delivery amongst women with obstetric anal sphincter injuries, and to determine if there was an association between antibiotic administration and reduced wound complications	Retrospective cohort	2016–2021	1550 43 women with perineal wound complication (4.1% of the 1050 women for whom there were postpartum encounter details for)	Spontaneous or assisted vaginal birth	3rd and 4th degree	1. Wound complication: if any encounter was associated with the following diagnosis codes: disruption of perineal obstetric wound (O90.1) or infection of obstetric surgical wound (O86.0)	1. BMI: BMI 25–29.9, BMI 30–34.9, BMI 35 or over (used in analysis), 2. Assisted vaginal birth, 3. Fourth degree tear, 4. Broad spectrum antibiotics *Model 2 estimates used because this is in‐keeping with current guidelines recommending prophylactic antibiotics for 3rd or 4th degree tears*	BMI, assisted vaginal birth, fourth degree laceration, broad spectrum antibiotics
Gommesen	2019	Denmark	To assess risk factors for perineal tears, wound infection and dehiscence among primiparous women	Prospective cohort study	2015–2018	603 23 women with perineal wound infection (5.8% of the 400 women with 2nd/3rd/4th‐degree tears) 61 women with perineal wound dehiscence (15.3% of the 400 women with a 2nd/3rd/4th degree tears)	Spontaneous or assisted vaginal birth	2nd degree or more severe tears	1. Wound infection: presence of purulent discharge or wound abscess 2. Wound dehiscence: gap of >0.5 cm between wound edges	1. Smoking, 2. Age 34 or older, 3. Diabetes, 4. Neonatal head circumference 35 cm or over, 5. Birthweight 3.5 kg or over, 6. BMI >35, 7. Assisted vaginal birth, 8. Group B streptococcus colonization, 9. Length of active birth >340 minutes, 10. Prolonged second stage >30 minutes, 11. Third/fourth‐degree tear, 12. Any antibiotics, 13. Episiotomy, 14. Per one additional year (maternal age), 15. Per centimeter increase to neonatal head circumference, 16. Per 100 g increase in neonatal birthweight, 17. Per 60 minute increase in active birth time, 18. Per 10 minute increase to second stage	Diabetes, episiotomy, assisted vaginal birth, BMI, duration of the second stage
Jallad	2016	USA	To determine risk factors associated with breakdown of perineal laceration repair	Case–control	2002–2015	288 included in case–control study 144 women with perineal wound breakdown (0.2% from the 68 839 women screened with a vaginal birth[infer denominator includes women with an intact perineum])	Spontaneous or assisted vaginal birth	2nd degree or more severe tears	1. Wound breakdown: wound breakdown requiring intervention, excluding those with superficial wound separation	1. Smoking, 2. Minority ethnic group, 3. BMI (unclear over which level), 4. Previous vaginal birth, 5. Assisted vaginal birth, 6. Third/fourth degree tear, 7. Episiotomy, 8. Repair by midwife, 9. Repair with chromic suture material	Age, BMI, race, previous vaginal birth, nulliparity, smoking, degree of laceration/episiotomy, assisted vaginal birth, provider performing repair, suture type
Kingsbury	2018	India	To assess the incidence and risk factors of peripartum wound dehiscence	Retrospective cohort/case–control analysis	2016	255 included in case–control analysis 86 women with perineal wound dehiscence (0.9% from the 10 088 women screened with a vaginal birth[infer denominator includes women with an intact perineum])	Spontaneous or assisted vaginal birth	Unspecified	1. Wound dehiscence: dehiscence requiring primary repair or management conservatively with dressings	1. Raised BMI >30, 2. Primiparity, 3. >3 vaginal examinations, 4. Anemia, 5. Induction of labour, 6. Prolonged rupture of membranes, 7. Assisted vaginal birth, 8. Meconium‐stained liquor	BMI >30, primiparity, assisted vaginal birth, meconium stained liquor, >3 vaginal examinations, anemia, prolonged rupture of membranes, induction of labour
Lallemant	2024	France	To describe short and mid‐term complications after primary OASI and to assess factors associated with complication occurrence	Retrospective cohort	2013–2021	61 833 2015 women with OASI complication (3.3%)	Spontaneous or assisted vaginal birth	3rd and 4th degree	1. Complications: Wound infection, suture breakdown, secondary perineal repair, fistula, sphincteroplasty, artificial anal sphincter, sacral nerve stimulation, colostomy	1. Assisted vaginal birth, 2. Obesity, 3. 4th degree tear, 4. Obstetric hematoma, 5. Age between 25 and 29 years old	List exceeds space however notably includes: age, prolonged second stage, assisted vaginal birth, type of perineal trauma, smoking, obesity, diabetes, parity (see Appendix S1 within original publication for full‐list^27^)
Lewicky‐Gaupp	2015	USA	To estimate the incidence of and risk factors for wound complications in women who sustain obstetric anal sphincter injuries	Prospective cohort	2011–2014	268 53 women with perineal wound infection (19.8%) 66 women with perineal wound breakdown (24.6%) 90 women overall with perineal wound complication (33.6%)	Spontaneous or assisted vaginal birth	3rd and 4th degree	1. Wound infection: three or more of heat, erythema, oedema, purulent discharge on examination 2. Wound breakdown: at least 1 cm breakdown 3. Wound complication: wound infection, breakdown, or both	1. Antibiotics for treatment for obstetric indication during admission, 2. Assisted vaginal birth	Age, assisted vaginal birth, degree of laceration, use of antibiotics
Meckes	2024	USA	To evaluate for characteristics associated with wound complications after OASI	Retrospective cohort	2020–2023	332 74 of women with perineal wound complication (22.3%)	Spontaneous or assisted vaginal birth	3rd and 4th degree	1. Wound complications: wound infection (purulent/foul smelling discharge, erythema or abscess formation), wound breakdown, wound dehiscence, need for additional antibiotics, need for surgical intervention	1. Older age, 2. Peripartum antibiotics (antibiotics given for group B streptococcus prophylaxis, perineal laceration repair or chorioamnionitis given within 24 hours of birth*). Included in earlier steps of model therefore not included in our results unless possible to include in sensitivity analysis*: 1. Assisted vaginal birth (able to include in sensitivity analysis only), 2 Episiotomy, 3. Type of perineal trauma, 4. Discharged on stool softener, 5. No previous vaginal births	Age, peripartum antibiotic use, no previous vaginal births, assisted vaginal birth, episiotomy, type of perineal trauma, bowel regimen on discharge
Propst	2023	USA	To evaluate the role of antibiotics on preventing wound complications following obstetric anal sphincter injury	Cohort with retrospective and prospective components	2018–2021	425 51 women with perineal wound complication (12.0%)	Spontaneous or assisted vaginal birth	3rd and 4th degree	1. Perineal wound complication: infection─ at least three of erythema, oedema, warmth, purulent discharge OR breakdown─ wound separation of layers deeper than the skin ≥1 cm OR rectovaginal fistula	1. No antibiotics given, 2. Age at time of birth (per one unit increase), 3. Non‐white ethnicity, 4. Fourth‐degree tear (compared to third), 5. Diabetes	Antibiotics use, age, race, degree of laceration, diabetes
Puissegur	2023	France	To identify risk factors associated with perineal wound breakdown	Retrospective case–control study	2010–2015	333 included in the case–control analysis 84 women with perineal wound breakdown (0.9% from the 9364 women screened with perineal trauma)	Spontaneous or assisted vaginal birth	Episiotomy/2nd degree or higher severity	1. Wound breakdown: separation of one or more stitches from the perineal suture	1. Primiparity, 2. Previous vaginal birth, 3. Assisted vaginal birth, 4. Prolonged second stage of labor (duration in minutes), 5. Perineal hematoma	Primiparity, history of vaginal birth, duration of second stage, assisted vaginal birth, perineal haematoma
Stock	2013	USA	To determine factors associated with perineal wound complications in women with obstetric anal sphincter injuries	Retrospective cohort	2005–2010	909 66 women with perineal wound complication (7.3%)	Spontaneous or assisted vaginal birth	3rd and 4th degree	1. Wound complication: infection, breakdown, packing, operative intervention or secondary repair	1. Smoking, 2. Per one additional BMI point, 3. Assisted vaginal birth, 4. Fourth‐degree tear, 5. Postpartum antibiotics, 6. Intrapartum antibiotics	Race, assisted vaginal birth, BMI, smoking, degree of tear, intrapartum antibiotics, postpartum antibiotics
Thongtip	2023	Thailand	To measure the incidence and assess the associated factors for perineal wound infection and dehiscence following vaginal delivery	Retrospective cross‐sectional	2018–2020	2589 44 women with perineal wound infection (1.7%)	Spontaneous or assisted vaginal birth	All requiring repair	1. Wound infection: purulent discharge and positive bacterial culture	1. Gestational hypertension, 2. Birthweight >3 kg, 3. >4 vaginal examinations, 4. Prophylactic antibiotics, 5. Provider: resident, 6. Provider: nurse student, 7. Provider: registered nurse for <5 years	Gestational hypertension, vaginal examination >4 times, birthweight, provider, prophylactic antibiotics
Wilkie	2018	USA	To investigate the risk factors for perineal wound breakdown after operative vaginal delivery	Retrospective cohort	2015–2016	529 14 women with perineal wound breakdown (2.6%)	Assisted vaginal birth	All	1. Wound breakdown: definition unclear	1. Forceps birth, 2. Episiotomy, 3. Narcotic use postpartum	Type of assisted birth, episiotomy, assisted vaginal birth indication, narcotic use postpartum
Williams	2006	USA	To identify risk factors associated with the breakdown of perineal laceration repair in the postpartum period	Retrospective case control	1995–2005	177 included in case–control study 59 women with perineal wound breakdown (0.4% from the 14 124 women screened with a vaginal birth [infer denominator includes women with an intact perineum])	Spontaneous or assisted vaginal birth	2nd, 3rd, 4th, episiotomy	1. Wound breakdown: Definition given for wound dehiscence: ‘complete separation of the mucosa of at least 50% of the length of the repair and/or deeper separation of the perineal body.’	1. Assisted vaginal birth with mediolateral episiotomy (used in assisted vaginal birth meta‐analysis), 2. Assisted vaginal birth with midline episiotomy, 3. Meconium‐stained liquor, 4. Prolonged second stage of labor >60 minutes, 5. Third/fourth‐degree tear	List exceeds space however notably includes: Age, BMI, previous vaginal birth, medical complications of pregnancy, episiotomy, higher order laceration, assisted vaginal birth, race, prolonged second stage of labour (See tab. 3 in original publication for full‐list^24^)
Zhang	2017	China	To investigate risk factors for episiotomy infection	Retrospective cohort	2012–2015	1200 30 women with perineal wound infection (2.5%)	Spontaneous, unclear whether also includes assisted vaginal birth	Episiotomy	1. Wound infection: definition unclear	1. Raised BMI >28, 2. >3 vaginal examinations, 3. Prolonged rupture of membranes, 4. Known reproductive tract infection, 5. Postoperative hospitalization time >5 days, 6. “complication with basic diseases” (unable to find details of which diseases are included)	BMI, number of vaginal examinations, postoperative hospitalization time, premature rupture of membranes, reproductive tract infections, complication with basic diseases (note this list does not appear exhaustive however unable to contact authors)

^a^ Note that authors have been emailed for clarification where there were uncertainties on the factors adjusted for. Where authors responded, this information has been included. Where we were unable to contact authors or where they could not provide clarification, the information presented is based only on what is available in the primary publication; thus, there may be minor inaccuracies.

Risk of bias in included studies was assessed using the QUIPS tool, which comprises six domains with each rated as low, moderate, or high risk of bias.^12^ In one study, risk of bias was low in two of six domains^25^; in three studies, risk of bias was low in three of six domains^15^, ^20^, ^27^; in eight studies, risk of bias was low in four of six domains^18^, ^19^, ^21^, ^23^, ^24^, ^26^, ^28^, ^29^; and in three studies, risk of bias was low in five domains^16^, ^17^, ^22^ (Table S1). Prognostic factor measurement and outcome measurement were the domains with the highest risk of bias across studies, with seven and 11 of 15 studies, respectively, having a moderate/high risk of bias for these domains.

### 3.1 Prognostic factor analysis

**FIGURE 2:**
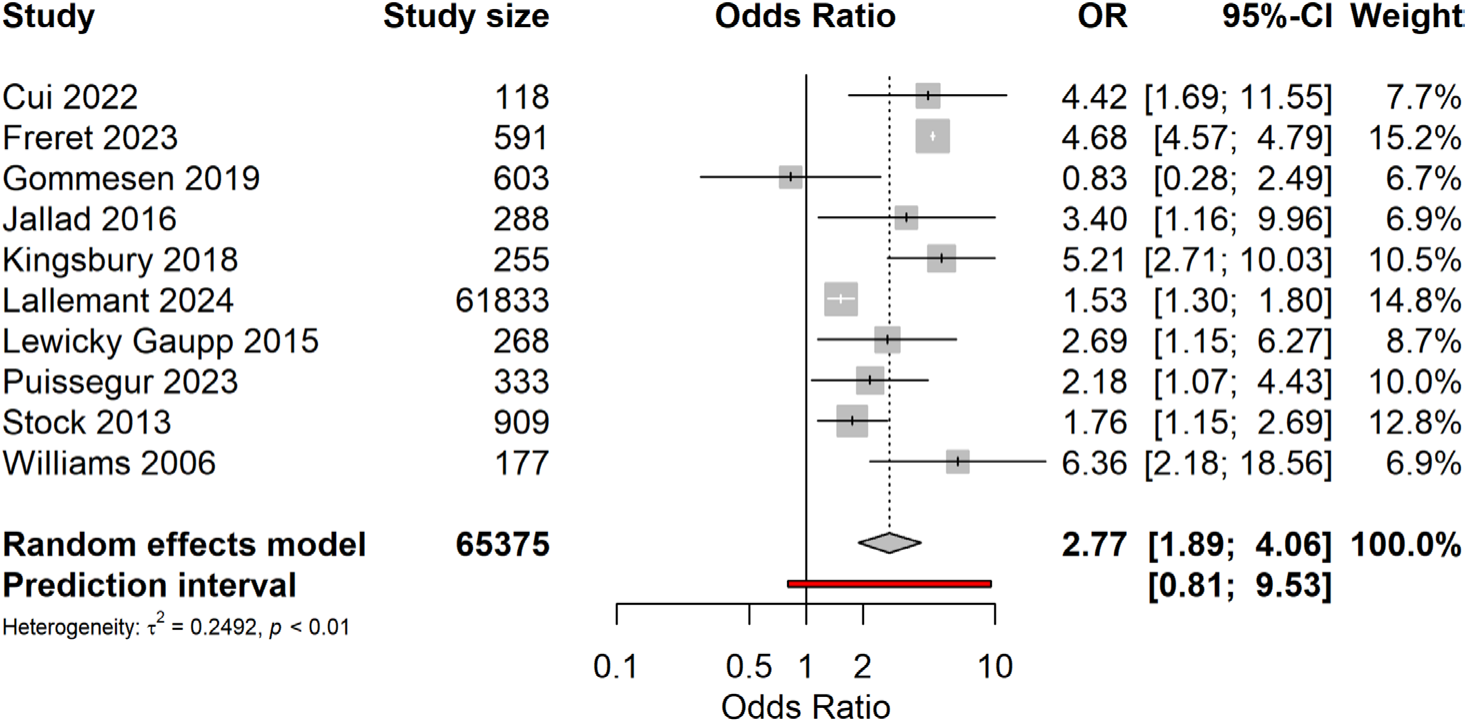
Forest plot to show the pooled odds ratio (OR) with 95% confidence interval (CI) for assisted vaginal birth as a risk factor for perineal wound complication. Inputted 95% CIs (where different to output): Cui 2022 95% CI 1.78–12.16, Gommesen 2019 95% CI 0.28–2.51, Jallad 2016 95% CI 1.20–10.30, Kingsbury 2018 95% CI 2.70–10.00, Lallemant 2024 95% CI 1.30–1.79, Lewicky‐Gaupp 2015 95% CI 1.12–6.08, Puissegur 2023 95% CI 1.07–4.41, Stock 2013 95% CI 1.15–2.68, Williams 2006 95% CI 2.18–18.57.

**FIGURE 3:**
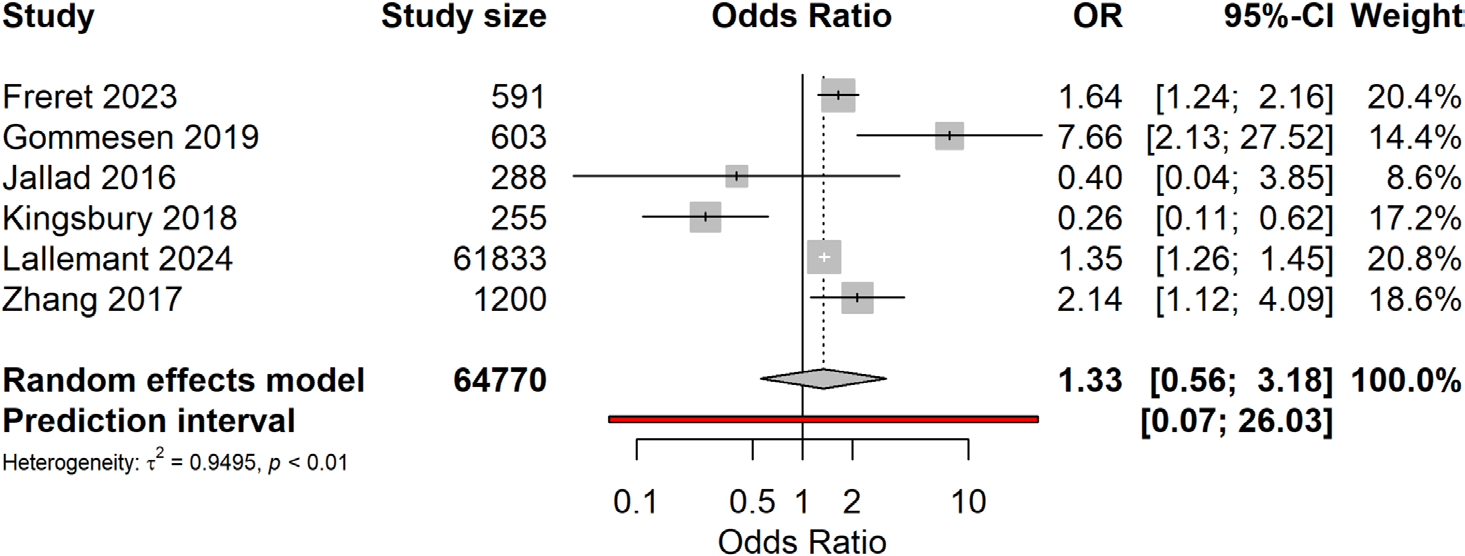
Forest plot to show the pooled odds ratio (OR) with 95% confidence interval (CI) for raised body mass index as a risk factor for perineal wound complication. Inputted 95% CIs (where different to output): Gommesen 2019 95% CI 2.13–27.50, Jallad 2016 95% CI 0.04–3.70, Kingsbury 2018 95% CI 0.11–0.63, Lallemant 2024 95% CI 1.50–1.72.

After meta‐analysis, assisted vaginal birth (10 studies, 65 375 women)^15^, ^16^, ^17^, ^18^, ^19^, ^20^, ^21^, ^24^, ^26^, ^27^ was a risk factor for perineal wound complication (OR 2.77, 95% CI 1.89–4.06 [prediction interval 0.81–9.53, *τ* ^2^ = 0.25]) (Figure 2). Raised body mass index (BMI) was not a significant risk factor for perineal wound complication (six studies, 64 770 women)^16^, ^17^, ^18^, ^25^, ^26^, ^27^ (OR 1.33, 95% CI 0.56–3.18 [prediction interval 0.07–26.03, *τ* ^2^ = 0.95]) (Figure 3).

**FIGURE 4:**
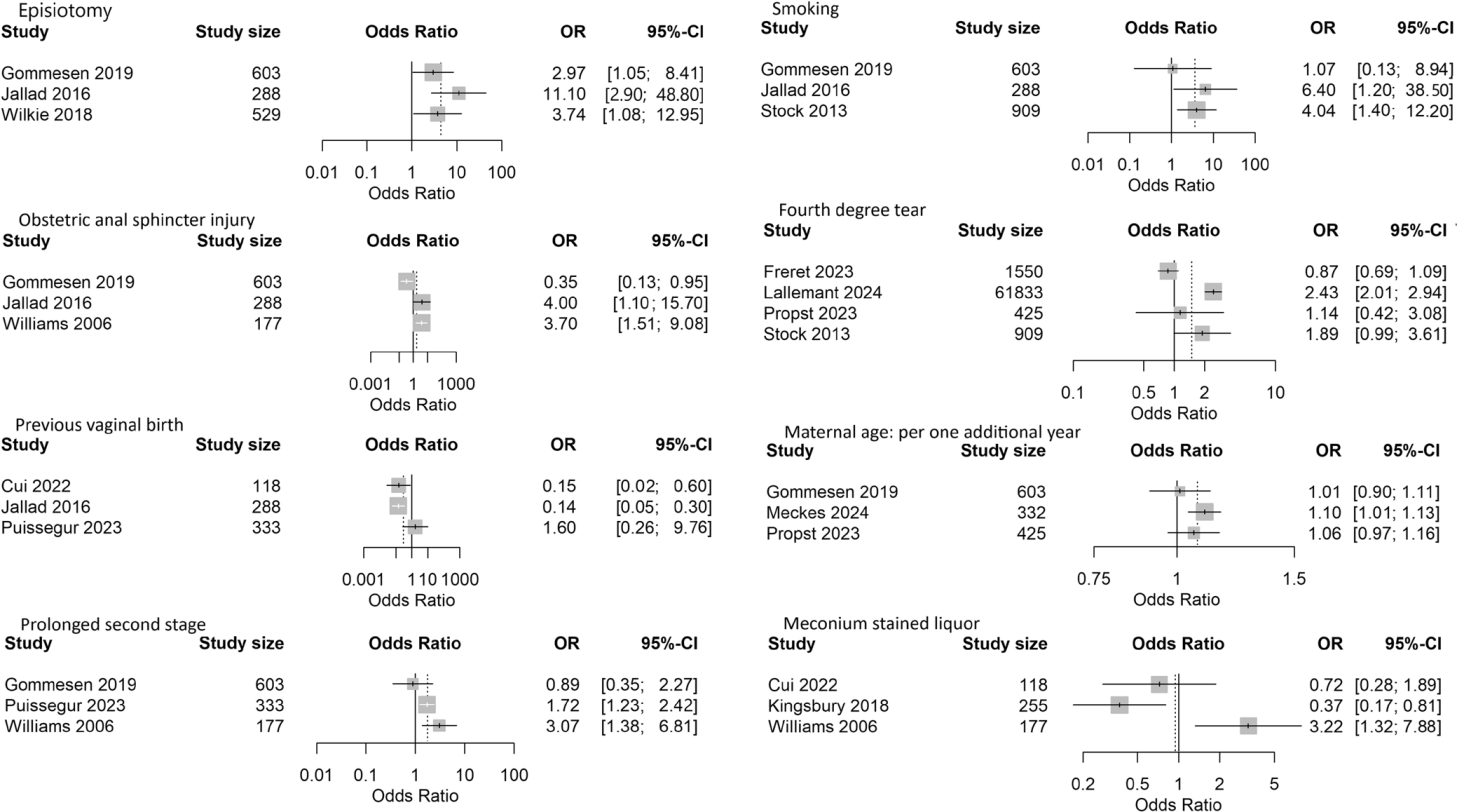
Forest plots demonstrating individual study results for the following factors: episiotomy, smoking, obstetric anal sphincter injury, fourth‐degree tear, previous vaginal birth, maternal age: per one additional year, prolonged second stage, meconium. Meta‐analysis was not performed as there were fewer than five studies eligible for each analysis.

Several other potential prognostic factors for perineal wound complication were investigated in three or four studies only; therefore, meta‐analysis was precluded. These included smoking, previous vaginal birth, prolonged second stage of labor, episiotomy, meconium‐stained liquor, obstetric anal sphincter injury, fourth‐degree tear (reference group: those with third‐degree tear) and increased maternal age (per one additional year). A graphical display of individual study results included for these prognostic factors can be found in Figure 4.

Smoking was investigated as a risk factor for perineal wound complication by Gommesen, Jallad, and Stock et al.^16^, ^17^, ^21^ Gommesen et al. conducted a prospective multi‐centre cohort study in Denmark, including 603 women. Here, smoking was not found to be an independent risk factor for perineal wound infection (aOR 1.07, 95% CI 0.13–8.94). In the latter two studies (Jallad et al.; a case control study including 288 women and Stock et al.; a retrospective cohort study including 909 women, both performed in the United States) smoking was found to be independently associated with perineal wound complications (aOR 6.40, 95% CI 1.20–38.50 and aOR 4.04, 95% CI 1.40–12.20, respectively). Prolonged second stage of labor was investigated in three studies: Gommesen, Puissegur (a French retrospective case–control study of 333 women) and Williams et al. (a retrospective case–control study of 177 women undertaken in the United States)^16^, ^20^, ^24^─the latter two studies found that prolonged second stage conferred increased risk of wound complication (aOR 0.89, 95% CI 0.35–2.27; aOR 1.72, 95% CI 1.23–2.42 and aOR 3.07, 95% CI 1.38–6.81, respectively). Episiotomy was investigated as a potential prognostic factor in Gommesen, Jallad, and Wilkie et al.^16^, ^17^, ^23^─all three studies found that episiotomy was an independent risk factor for perineal wound complication (aOR 2.97, 95% CI 1.05–8.41; aOR 11.1, 95% CI 2.9–48.8; aOR 3.74, 95% CI 1.08–12.95, respectively).

Primiparity was investigated as a risk factor for perineal wound complication in two studies.^18^, ^20^ Kingsbury et al., a retrospective study including 255 women in India, found that primiparity was an independent risk factor for perineal wound dehiscence (aOR 2.86 95% CI 1.23–6.66).^18^ Puissegur et al., contrastingly, found that primiparity was not a statistically significant risk factor for perineal wound breakdown (aOR 1.87 95% CI 0.38–9.25).^20^

Prolonged rupture of membranes was investigated as a potential risk factor in two studies, Kingsbury et al. (characteristics as above) and Zhang et al., a Chinese retrospective cohort of 1200 women who underwent episiotomy.^18^, ^25^ The former study found that prolonged rupture of membranes was not a statistically significant risk factor (aOR 0.53, 95% CI 0.21–1.33); however, the latter found an independent association of prolonged rupture of membranes with an increased risk of episiotomy infection (aOR 1.63 95% CI 1.06–2.50). Minority ethnic group was considered a risk factor for wound complication in two studies, Propst et al. and Jallad et al., with neither study finding a significant effect (aOR 0.61, 95% CI 0.25–1.49 and aOR 3.2, 95% CI 0.8–16.4, respectively).^17^, ^29^ Propst et al. and Gommesen et al. investigated the effect of diabetes on wound complication, with neither study finding a statistically significant effect (aOR 1.75, 95% CI 0.60–5.13, aOR 0.70 95% CI 0.08–5.91, respectively). Additional potential risk factors documented in two studies included known reproductive tract infection, e.g., Group B Streptococcus, induction of labor, BMI (per one additional BMI point), perineal hematoma, and more than three vaginal examinations─ results are compiled in Table 2.

**TABLE 2:** Adjusted odds ratios from individual studies for prognostic factors not displayed in forest plots (prognostic factor recorded in two or less studies).

		Factors investigated in two studies with estimates (adjusted odds ratios [aOR] and 95% confidence intervals [CI])									Factors investigated by a single study with estimates^a^ (aOR and 95% CI)
Study	Outcome	Reproductive tract infection (e.g. Group B Streptococcus[GBS])	Primiparity	Per one additional BMI point	Diabetes	Minority ethnic group	Induction of labour	Prolonged rupture of membranes	>3 vaginal examinations	Perineal haematoma	
Cui 2022	Wound breakdown						aOR 1.05 95% CI 0.45–2.89				Labor augmentation: aOR 0.37 95% CI 0.029–5.57, postpartum hemorrhage >500 mL: aOR 10.44, 95% CI 1.87–195.8, working years for delivery provider 1–3 years: aOR 1.019 95% CI 0.36–2.93, working years for delivery provider 3–5 years: aOR 0.44; 95% CI 0.11–1.56, working years for delivery provider 5–10 years: aOR 0.27, 95% CI 0.07–0.85
Freret 2023	Wound complication										BMI 25–29.9: aOR 0.47, 95% CI 0.26–0.83, BMI 30–34.9: aOR 0.97, 95% CI 0.25–3.73
Gommesen 2019	Wound infection	aOR 1.37 95% CI 0.16–11.5^b^		aOR 1.12 95% CI 1.04–1.22	aOR 0.70 95% CI 0.08–5.91						Age 34 or older: aOR 1.57 95% CI 0.24–10.3, neonatal head circumference 35 cm or over: aOR 1.10 95% CI 0.45–2.67, birthweight >3.5 kg: aOR 0.37 95% CI 0.15–0.96, length of active birth >340 minutes: aOR 1.16 95% CI 0.47–2.86, any antibiotics: aOR 0.52 95% CI 0.18–1.50, per centimeter increase to neonatal head circumference: aOR 1.02, 95% CI 0.78–1.35, per 100 g increase in neonatal birthweight: aOR 0.91, 95% CI 0.82–1.01, per 60 minute increase in active birth time: aOR 1.00, 95% CI 0.91–1.10, per 10 minute increase to second stage: aOR 1.01, 95% CI 0.86–1.18
Gommesen 2019	Wound dehiscence	aOR 1.98 95% CI 0.51–7.68^b^		aOR 1.03 95% CI 0.97–1.09	aOR 1.32 95% CI 0.35–5.01						Episiotomy: aOR 1.64 95% CI 0.79–3.43, Third/fourth‐degree tear: aOR 0.64 95% CI 0.36–1.15, Smoking: aOR 0.34 95% CI 0.04–2.69, BMI >35: aOR 3.46 95% CI 1.10–10.9, Assisted vaginal birth: aOR 0.56 95% CI 0.27–1.17, age 34 or older: aOR 3.36 95% CI 0.79–14.3, per one additional year maternal age: aOR 0.99 95% CI 0.92–1.06, neonatal head circumference 35 cm or over: aOR 1.26 95% CI 0.71–2.25, birthweight >3.5 kg: aOR 0.59 95% CI 0.33–1.05, length of active birth >340 minutes: aOR 0.94 95% CI 0.53–1.66, any antibiotics: aOR 0.32 95% CI 0.15–0.70, per centimeter increase to neonatal head circumference: aOR 1.02, 95% CI 0.85–1.21, per 100 g increase in neonatal birthweight: aOR 0.98, 95% CI 0.93–1.04, per 60 minute increase in active birth time: aOR 0.95, 95% CI 0.89–1.02, per 10 minute increase to second stage: aOR 1.10, 95% CI 1.00–1.21, prolonged second stage of labor: aOR 1.53 0.85–2.76
Jallad 2016	Wound breakdown					aOR 3.2 95% CI 0.8–16.4					Repair by midwife: aOR 4.7 95% CI 1.5–15.8, chromic suture material: aOR 3.9 95% CI 1.6–9.8
Kingsbury 2018	Wound dehiscence		aOR 2.86 95% CI 1.23–6.66				aOR 1.35, 95% CI 0.71–2.56	aOR 0.53, 95% CI 0.21–1.33	aOR 0.73, 95% CI 0.26–2.08		Anemia: aOR 0.38, 95% CI 0.15–0.98
Lallemant 2024	Complication composite									aOR 1.81, 95% CI 1.22–2.67	Age 25–29 years old: aOR 1.27, 95% CI 1–1.61
Lewicky‐Gaupp 2015	Wound breakdown										Assisted vaginal birth: aOR 2.29, 95% CI 1.11–4.75
Lewicky‐Gaupp 2015	Wound complication composite: wound infection or breakdown										Assisted vaginal birth: aOR 2.54, 95% CI 1.32–4.87, Antibiotics for treatment for obstetric indication during admission: aOR 0.50, 95% CI 0.27–0.94
Meckes 2024	Wound complication composite										Peripartum antibiotics: aOR, 0.57, 95% CI, 0.33–0.97
Propst 2023	Wound complication composite				aOR 1.75, 95% CI 0.60–5.13	aOR 0.61, 95% CI 0.25–1.49					No antibiotics: aOR 0.86, 95% CI 0.40–1.83
Puissegur 2023	Wound breakdown		aOR 1.87 95% CI 0.38–9.25							aOR 1.83, 95% CI 0.66–5.04	
Stock 2013	Wound complication composite			aOR 1.06, 95% CI 1.01–1.12							Postpartum antibiotics: aOR 2.46, 95% CI 1.11–5.63, intrapartum antibiotics: aOR 0.29 95% CI 0.14–0.59
Thongtip 2023	Wound infection										Gestational hypertension: aOR 3.77 95% CI 1.28–11.12, birthweight >3 kg: aOR 4.28 95% CI 1.89–9.7, >4 vaginal examinations: aOR 4.21 95% CI 2.29–7.73, prophylactic antibiotics: aOR 0.29, 95% CI 0.1–0.82, provider‐ registered nurse for <5 years: aOR 2.66, 95% CI 1.26–5.6, provider‐ nurse student: aOR 2.45, 95% CI 0.99–6.03, provider‐ resident: aOR 3.44, 95% CI 0.8–14.8
Wilkie 2018	Wound breakdown										Forceps assisted birth: aOR 6.73 95% CI 1.99–22.78, narcotic use postpartum: aOR 21.29 95% CI 5.43–83.47
Williams 2006	Wound breakdown										Assisted vaginal birth with midline episiotomy aOR 0.15, 95% CI 0.02–1.45
Zhang 2017	Wound infection	aOR 1.865 95% CI 1.053–3.300^c^						aOR 1.627 95% CI 1.059–2.502	aOR 1.85 95% CI 1.162–2.945		Post‐operative hospitalization time >5 days: aOR 2.62 95% CI 1.363–5.032, complication with basic diseases: aOR 2.022, 95% CI 1.220–3.350

^a^ For Gommesen and Lewicky‐Gaupp— where risk factor adjusted estimates were provided for >1 relevant outcome, estimates for risk factors recorded for the additional outcome (not the outcome included preferentially in meta‐analysis) are included in this column if not included elsewhere in the table or figures.

^b^ Gommesen et al. investigated GBS as the risk factor.

^c^ Zhang et al. investigated any reproductive tract infection as the risk factor.

Several other factors were investigated in only one study. These included gestational hypertension, increased neonatal head circumference, postpartum hemorrhage, and maternal anemia (in addition to further risk factors as detailed in Table 2). The individual study results for each investigated prognostic factor where estimates have not been graphically displayed in forest plots are available in Table 2.

### 3.2 Sensitivity and subgroup analyses

Due to differences in the definition of wound complication in the study by Lallemant et al.,^27^ which included a small number of patients with broader complications of perineal trauma aside from those specific to wound healing, sensitivity analyses were performed excluding this study. For assisted vaginal birth, this remained independently prognostic of wound complication with the exclusion of the Lallemant study (OR 3.08, 95% CI 2.10–4.53 [prediction interval 0.97–9.81, *τ* ^2^ = 0.20]) (Figure S1). For raised BMI, there remained no effect on the rates of wound complication (OR 1.31, 95% CI 0.42–4.12 [prediction interval 0.02–85.48, *τ* ^2^ = 1.38]) after exclusion of Lallemant et al. (Figure S2).

A further sensitivity analysis is available for assisted vaginal birth, including an additional unpublished estimate from the earlier steps of the logistic regression model from Meckes et al. (Figure S3).^28^ Figure S4 demonstrates a sensitivity analysis for assisted vaginal birth, with removal of studies where results were not clearly adjusted for perineal trauma type (where studies included women with a range of perineal trauma types). A similar sensitivity analysis could not be performed for the raised BMI analysis, as removal of studies that had not clearly adjusted for perineal trauma type resulted in an inadequate number of studies for meta‐analysis. Overall, for both further sensitivity analyses of assisted vaginal birth, the direction of results was unchanged compared to the main analysis. Subgroup analysis by type of perineal trauma was possible for assisted vaginal birth only, which demonstrated no significant subgroup differences (*p* = 0.27) (Figure S5).

## 4 DISCUSSION

Our meta‐analysis showed that assisted vaginal birth was an independent risk factor for perineal wound complication (OR 2.77, 95% CI 1.89–4.06); whereas raised BMI was not found to be a significant risk factor. The remaining potential prognostic factors were each investigated in four or fewer studies and therefore meta‐analysis could not be performed. The sensitivity analysis for assisted vaginal birth, including only studies that clearly utilized adjustment for perineal trauma type, demonstrated that the magnitude and direction of the pooled result remained unchanged. This suggests that assisted vaginal birth is a risk factor for perineal wound complication independently from the type of perineal trauma. Reasons for this may include increased instrumentation and digitation of the vaginal tract with assisted vaginal birth, resulting in the introduction of infection. There is also the potential for micro‐disruption to the vaginal tissue from the use of forceps or ventouse, which may not necessarily be reflected in the broad perineal trauma types.

Interestingly, all individual studies included in the analysis for assisted vaginal birth found that it was a statistically significant prognostic factor for perineal wound complication, apart from Gommesen et al.^16^ Gommesen et al. included a very small number of women who developed perineal wound infection (*n* = 23), of whom only five women experienced assisted vaginal birth. This may therefore limit the generalizability of the results from this individual study.

It is of note that episiotomy is also more common among women who have undergone assisted vaginal birth. Whilst we were unable to perform meta‐analysis for episiotomy due to an insufficient number of studies, the three studies which investigated this factor each found that episiotomy was an independent risk factor for perineal wound complication (each of these three studies were adjusted for the effects of assisted vaginal birth). It is therefore a possibility, albeit not one that can be confidently concluded from this work, that both assisted vaginal birth and episiotomy independently increase the risk of perineal wound complication, meaning that women who undergo both would have a high risk of wound complication. However, this suggestion could not be corroborated with the assisted vaginal birth prognostic factor meta‐analysis subgrouped by type of perineal trauma, which found no difference in the prognostic effect of assisted vaginal birth between perineal trauma types. This analysis was however significantly limited by the designs of the included studies–notably there was no study in this analysis which included only women with episiotomy (distinct from other tear types).

To our knowledge, this is the first systematic review investigating prognostic factors for childbirth‐related perineal trauma wound complications. It is crucial we are able to identify which women may be more at risk of wound complications to target interventions aimed at reducing subsequent wound‐related morbidity—this systematic review provides the building blocks for doing so. Through using a broad range of search terms in several databases without language or date restrictions, our review incorporates a comprehensive set of primary studies. By pooling aORs we are also able to identify prognostic factors that persist independently of others, increasing the direct clinical relevance of the study. Whilst the inclusion of studies that calculated unadjusted estimates would lead to an increased amount of information on which to base conclusions, meta‐analysis of unadjusted and adjusted results combined or of unadjusted results alone would provide little meaningful information, as it would be unclear which prognostic factors persisted independently. In terms of direct clinical applications, we highlight a group that could benefit from increased monitoring in the postpartum period. It is imperative that care pathways are developed utilizing information on who is most at risk of developing wound complications. This would mean women and healthcare professionals are not only clear on appropriate interventions (e.g., prophylactic antibiotics for those who have undergone assisted vaginal birth, which our results support) but also that women at higher risk of wound complications receive specialist postpartum care with increased monitoring for wound problems.

Additionally, where meta‐analysis was possible, measures of heterogeneity were generally high. This demonstrates that the expected range of true effects across similar studies is large and that the effect of the predictive factor could in fact be in the opposite direction to the point estimate.^14^ The large prediction intervals will be in part due to the small number of primary studies available for meta‐analysis for these factors, in addition to differences between studies in definitions of both prognostic factors and what constitutes a wound complication.

Included studies investigated a range of possible prognostic factors, some of which were poorly defined or where the definition varied too greatly from other studies to be appropriately included in meta‐analysis. Similarly, authors investigated a range of wound‐related outcomes, including infection, wound dehiscence, and composite wound complication outcomes, which had varying definitions between studies. While we made a practical decision to perform meta‐analysis of prognostic factors for the broad outcome of wound complication, therefore encompassing any of the wound‐related adverse outcomes, this must be taken into account when interpreting our results. If there were a greater number of eligible studies with consistent definitions of adverse wound outcomes, then ideally prognostic factors for each different wound complication would be investigated separately, e.g., those for wound infection and those for wound dehiscence—unfortunately, within the confines of the existing primary research, this was not possible.

Notably, we made the decision to include the large retrospective cohort study by Lallemant et al. in our systematic review,^27^ which included complications after OASI for up to 2 years post‐birth and determined the risk factors for these complications as a composite outcome. The majority of patients who experienced a complication in this study developed perineal wound breakdown, wound infection, fistula, or required secondary repair; therefore, in keeping with the scope of our systematic review. However, a minority (~11%) developed other complications such as the need for sphincteroplasty or colostomy. As the majority of cases of complication were in keeping with our systematic review aims and the study provided such a valuable source of information, we made the decision to include this study. However, because of differences in outcome, sensitivity analyses were also performed excluding Lallemant et al. Sensitivity analyses importantly showed no difference in the direction of effect or statistical significance for either assisted vaginal birth or raised BMI compared to the original analysis containing all studies.

Limited studies were available investigating prognostic factors for perineal wound complications; therefore, for the majority of prognostic factors, we were not able to perform meta‐analysis. For example, the impact of diabetes was investigated only by two studies, precluding meta‐analysis for this factor. Similarly, analysis of the impact of other pregnancy‐related or existing health problems was lacking across studies. Whilst assisted vaginal birth was found to be a risk factor for perineal wound complication, adjusted estimates for ventouse/forceps separately were not available in the majority of studies, precluding meta‐analysis. Notably, Wilkie et al. found that forceps birth specifically was an independent risk factor for perineal wound complication compared to ventouse.^23^ Additionally, as there were limited studies available for analysis investigating women with non‐OASI perineal trauma only, the subgroup analysis for assisted vaginal birth by type of perineal trauma tells us limited information about this group of women specifically. In future studies, it would be important to ascertain whether certain prognostic factors persist across all perineal trauma types or whether they are specific to certain categories of tear.

In terms of prognostic factors for postnatal infection overall (including other forms of infection in additional to perineal wound infection, such as endometritis, urinary tract infection, sepsis of unknown origin etc.) there has been the recent secondary analysis of the ANODE (Prophylactic Antibiotics in the Prevention of Infection after Operative Vaginal Delivery) randomized controlled trial.^30^, ^31^ This secondary analysis by Humphreys et al. aimed to determine the factors associated with a composite infection outcome after assisted vaginal birth by utilizing data from 3225 women from the primary ANODE study.^30^, ^31^ It is important to note that we could not include this study in our systematic review, as the risk factors were determined for a composite infection outcome, as opposed to a specific perineal wound infection outcome. In their adjusted analysis, they found that episiotomy, forceps birth (in comparison to ventouse) and primiparity were risk factors for postnatal infection. Notably, these are broadly consistent with our systematic review findings. Humphreys et al. also found that BMI ≥25 kg/m^2^ was also associated with an increased risk of infection; however, interestingly, this did not persist for BMI ≥30 kg/m^2^.

## 5 CONCLUSION

Assisted vaginal birth is a significant risk factor for perineal wound complication. Further research is vital to ensure that the full spectrum of prognostic factors is identified, utilizing robust and well powered studies. By identifying prognostic factors for perineal wound complications, interventions aimed at reducing postnatal perineal wound complications can prioritize these groups, ultimately leading to improved outcomes amongst those most at risk.

## AUTHOR CONTRIBUTIONS

R.M.: conceptualization, data curation, formal analysis, methodology, investigation, writing original draft, writing‐ review and editing. T.B.: investigation, data curation. A.S.: methodology, supervision, writing‐ review and editing. K.M.: supervision, funding acquisition, writing, review and editing. V.H.M.: investigation, data curation, supervision, writing, review and editing. CHAPTER group: funding acquisition, writing, review and editing.

## FUNDING INFORMATION

This project is funded by the National Institute for Health and Care Research (NIHR), grant number NIHR202869. The views expressed are those of the authors and not necessarily those of NIHR or the Department of Health and Social Care. AS is supported by the NIHR Birmingham BRC.

## CONFLICT OF INTEREST STATEMENT

The authors report no conflicts of interest.

## Supporting information


**Appendix S1.** Database search terms.

**Figure S1.** Forest plot to show the pooled odds ratio (OR) with 95% confidence interval (CI) for assisted vaginal birth as a risk factor for perineal wound complication. Sensitivity analysis excluding Lallemant 2024.

**Figure S2.** Forest plot to show the pooled odds ratio (OR) with 95% confidence interval (CI) for raised body mass index as a risk factor for perineal wound complication. Sensitivity analysis excluding Lallemant 2024.

**Figure S3.** Forest plot to show the pooled odds ratio (OR) with 95% confidence interval (CI) for assisted vaginal birth as a risk factor for perineal wound complication. Sensitivity analysis including unpublished estimates from earlier steps of logistic regression model by Meckes 2024.

**Figure S4.** Forest plot to show the pooled odds ratio (OR) with 95% confidence interval (CI) for assisted vaginal birth as a risk factor for perineal wound complication. Sensitivity analysis including only studies that state adjustment was made for type of perineal trauma.

**Figure S5.** Forest plot to show the pooled odds ratio (OR) with 95% confidence interval (CI) for assisted vaginal birth as a risk factor for perineal wound complication, with subgroups by type of perineal trauma.

**Table S1.** Risk of bias assessments for included studies.

## Data Availability

The data that supports the findings of this study are available in the supplementary material of this article.

## References

[1] Listen to Mums: ending the postcode lottery on perinatal care. A report by the all‐party parliamentary group on birth trauma. Accessed May 12, 2025. https://www.theo‐clarke.org.uk/birth‐trauma‐report

[2] Smith LA , Price N , Simonite V , Burns EE . Incidence of and risk factors for perineal trauma: a prospective observational study. BMC Pregnancy Childbirth. 2013;13:59. 23497085 10.1186/1471-2393-13-59 PMC3599825

[3] Lindberg I , Persson M , Nilsson M , Uustal E , Lindqvist M . “Taken by surprise”—Women's experiences of the first eight weeks after a second degree perineal tear at childbirth. Midwifery. 2020;87:102748. 32454376 10.1016/j.midw.2020.102748

[4] Baumann S , Staudt A , Horesh D , Eberhard‐Gran M , Garthus‐Niegel S , Horsch A . Perineal tear and childbirth‐related posttraumatic stress: a prospective cohort study. Acta Psychiatr Scand. 2024;150(5):446‐457. 37550260 10.1111/acps.13595

[5] Huber M , Malers E , Tunón K . Pelvic floor dysfunction one year after first childbirth in relation to perineal tear severity. Sci Rep. 2021;11:12560. 34131194 10.1038/s41598-021-91799-8 PMC8206367

[6] Trovik J , Thornhill HF , Kiserud T . Incidence of obstetric fistula in Norway: a population‐based prospective cohort study. Acta Obstet Gynecol Scand. 2016;95:405‐410. 26713965 10.1111/aogs.12845

[7] Jones K , Webb S , Manresa M , Hodgetts‐Morton V , Morris RK . The incidence of wound infection and dehiscence following childbirth‐related perineal trauma: a systematic review of the evidence. Eur J Obstet Gynecol Reprod Biol. 2019;240:1‐8. 31202973 10.1016/j.ejogrb.2019.05.038

[8] RCOG . The Management of Third and Fourth Degree Tears. Green top guideline No 29. Accessed May 12, 2025. https://www.rcog.org.uk/media/5jeb5hzu/gtg‐29.pdf

[9] The Chapter Study—Childbirth Acquired Perineal Trauma Study. Accessed May 12, 2025. https://www.birmingham.ac.uk/research/applied‐health/research/chapter‐study

[10] PROSPERO. A series of systematic reviews exploring childbirth related perineal trauma. Accessed May 12, 2025. https://www.crd.york.ac.uk/PROSPERO/view/CRD42023458738

[11] Preferred reporting items for systematic reviews and meta‐analyses (PRISMA). Accessed May 12, 2025. https://www.prisma‐statement.org/prisma‐2020‐checklist

[12] Hayden JA , van der Windt DA , Cartwright JL , Côté P , Bombardier C . Assessing bias in studies of prognostic factors. Ann Intern Med. 2013;158:280‐286. 23420236 10.7326/0003-4819-158-4-201302190-00009

[13] Riley RD , Moons KGM , Snell KIE , et al. A guide to systematic review and meta‐analysis of prognostic factor studies. BMJ. 2019;364:k4597. 30700442 10.1136/bmj.k4597

[14] IntHout J , Ioannidis JP , Rovers MM , Goeman JJ . Plea for routinely presenting prediction intervals in meta‐analysis. BMJ Open. 2016;6:e010247. 10.1136/bmjopen-2015-010247 PMC4947751 27406637

[15] Cui L , Zhang H , Li L , Wang CC . Risk factors associated with breakdown of perineal laceration repair after vaginal birth. J Obstet Gynaecol. 2022;42:1543‐1546. 35166164 10.1080/01443615.2022.2033961

[16] Gommesen D , Nohr EA , Drue HC , Qvist N , Rasch V . Obstetric perineal tears: risk factors, wound infection and dehiscence: a prospective cohort study. Arch Gynecol Obstet. 2019;300:67‐77. 31004221 10.1007/s00404-019-05165-1

[17] Jallad K , Steele SE , Barber MD . Breakdown of perineal laceration repair after vaginal delivery: a case‐control study. Female Pelvic Med Reconstr Surg. 2016;22(4):276‐279. 27054788 10.1097/SPV.0000000000000274

[18] Kingsbury B , Rathore S , Chelliah H , Londhe V , Benjamin SJ , Mathews J . Risk factors for peripartum wound dehiscence. Clin Diagn Res. 2018;12:QC08‐QC11.

[19] Lewicky‐Gaupp C , Leader‐Cramer A , Johnson LL , Kenton K , Gossett DR . Wound complications after obstetric anal sphincter injuries. Obstet Gynecol. 2015;125:1088‐1093. 25932836 10.1097/AOG.0000000000000833

[20] Puissegur A , Accoceberry M , Rouzaire M , et al. Risk factors for perineal wound breakdown in early postpartum: a retrospective case‐control study. J Clin Med. 2023;12(8):3036. 37109371 10.3390/jcm12083036 PMC10146046

[21] Stock L , Basham E , Gossett DR , Lewicky‐Gaupp C . Factors associated with wound complications in women with obstetric anal sphincter injuries (OASIS). Am J Obstet Gynecol. 2013;208:327.e1‐327.e6. 10.1016/j.ajog.2012.12.025 23262251

[22] Thongtip N , Srilar A , Luengmettakul J . The incidence and associated factors of perineal wound infection following vaginal delivery in Charoenkrung Pracharak hospital, Bangkok, Thailand. Thai J Obstet Gynaecol. 2023;31:145‐153.

[23] Wilkie GL , Saadeh M , Robinson JN , Little SE . Risk factors for poor perineal outcome after operative vaginal delivery. J Perinatol. 2018;38:1625‐1630. 30337732 10.1038/s41372-018-0252-2

[24] Williams MK , Chames MC . Risk factors for the breakdown of perineal laceration repair after vaginal delivery. Am J Obstet Gynecol. 2006;195:755‐759. 16949409 10.1016/j.ajog.2006.06.085

[25] Zhang H , Han S . Risk factors and preventive measures for postoperative infection in episiotomy of puerperal. Biomed Res. 2017;28(20):8857‐8861.

[26] Freret TS , James K , Kaimal AJ . Antibiotic administration and wound complications after obstetric anal sphincter injuries. Am J Obstet Gynecol MFM. 2023;5:100883. 36736824 10.1016/j.ajogmf.2023.100883

[27] Lallemant M , Bartolo S , Ghesquiere L , et al. Midterm complications after primary obstetrical anal sphincter injury repair in France. BMC Pregnancy Childbirth. 2024;24:539. 39143527 10.1186/s12884-024-06691-w PMC11325760

[28] Meckes NA , Toal CT , Wang L , Giugale LE . Risk factors for wound complications after obstetric anal sphincter injury. Urogynecology (Phila). 2025;31:405‐411. 39744876 10.1097/SPV.0000000000001642

[29] Propst K , Yao M , Hickman LC . Impact of peripartum antibiotics on wound complications in women with obstetric anal sphincter injury. Int J Gynaecol Obstet. 2023;161:491‐498. 36306399 10.1002/ijgo.14530

[30] Knight M , Chiocchia V , Partlett C , et al. Prophylactic antibiotics in the prevention of infection after operative vaginal delivery (ANODE): a multicentre randomised controlled trial. Lancet. 2019;393:2395‐2403. 31097213 10.1016/S0140-6736(19)30773-1 PMC6584562

[31] Humphreys ABC , Linsell L , Knight M . Factors associated with infection after operative vaginal birth—a secondary analysis of a randomized controlled trial of prophylactic antibiotics for the prevention of infection following operative vaginal birth. Am J Obstet Gynecol. 2023;228:328.e1‐328.e11. 10.1016/j.ajog.2022.08.037 36027955

