# Supplementary material for: Prognostic factors for wound complications after childbirth‐related perineal trauma: A systematic review and meta‐analysis
Source: Acta Obstet Gynecol Scand. 2025 Aug 20;105(7):1247–64. doi: 10.1111/aogs.70041 (PMC13308966; doi:10.1111/aogs.70041)
Supplement: Supplementary file 1 — Appendix S1. Database search terms. [file AOGS-105-1247-s002.pdf]

Search terms risk factor for complications after perineal trauma SR

Ovid MEDLINE(R) and In-Process, In-Data-Review & Other Non-Indexed Citations 1946 to April 17, 2024 Searched 18th April 2024 (then updated to December 2024)

# ▲	Searches
1	*Anal Canal/in
2	exp Obstetric Labor Complications/ or exp Parturition/ or (birth or childbirth or labo?r).ab,kw,ti.
3	1 and 2
4	(obstetric* and anal and sphincter and (tear* or injur* or trauma or lacerat* or wound* or rupture*)).ab,kw,ti.
5	(OASIS or OASI).ab,kw,ti.
6	((third or 3rd or 4th or fourth) and degree and (perineal or perineum or vagina*) and (tear* or injur* or trauma or lacerat* or wound* or rupture*)).ab,kw,ti.
7	*Perineum/in [Injuries]
8	exp Perineum/
9	exp "Wounds and Injuries"/
10	8 and 9
11	((perineal or perineum or vagina*) and (tear* or injur* or trauma or lacerat* or wound* or rupture*)).ab,kw,ti.
12	2 and (7 or 10 or 11)
13	((childbirth or birth or labo?r or obstetric* or deliver*) and related and (perineal or perineum or vagina*) and (tear* or injur* or trauma or lacerat* or wound* or rupture*)).ab,kw,ti.
14	exp Episiotomy/
15	(episiotomy or episiotomies).ab,kw,ti.
16	14 or 15
17	4 or 5 or 6
18	3 or 17 or 12 or 13 or 16
19	exp Infections/
20	exp Surgical Wound Dehiscence/
21	wound breakdown.mp.
22	exp Surgical Wound Infection/ or exp Postoperative Complications/
23	exp Wound Infection/
24	wound infection.mp.
25	wound dehiscence.mp.
26	19 or 20 or 21 or 22 or 23 or 24 or 25
27	risk factor.mp. or exp Risk Factors/
28	prognostic factor.mp. or exp Prognosis/
29	27 or 28
30	18 and 26 and 29

Save Remove Combine with: AND OR

Embase 1974 to 2024 April 17 Searched 18th April 2024 (then updated to December 2024)

Search History (30)

# ▲	Searches
1	exp anus injury/
2	(exp anus sphincter/ or exp anal canal/) and exp injury/
3	exp Obstetric Labor Complications/ or exp Parturition/ or (birth or childbirth or labo?r or peri-partum or post-partum).ab,kw,ti.
4	(1 or 2) and 3
5	(obstetric* and anal and sphincter and (tear* or injur* or trauma or lacerat* or wound* or rupture*)).ab,kw,ti.
6	(OASIS or OASI).ab,kw,ti.
7	((third or 3rd or 4th or fourth) and degree and (perineal or perineum or vagina*) and (tear* or injur* or trauma or lacerat* or wound* or rupture*)).ab,kw,ti.
8	exp perineum/ or exp perineum muscle/
9	exp injury/
10	8 and 9
11	((perineal or perineum or vagina*) and (tear* or injur* or trauma or lacerat* or wound* or rupture*)).ab,kw,ti.
12	10 or 11
13	((childbirth or birth or labo?r or deliver*) and related and (perineal or perineum or vagina*) and (tear* or injur* or trauma or lacerat* or wound* or rupture*)).ab,kw,ti.
14	exp Episiotomy/
15	(episiotomy or episiotomies).ab,kw,ti.
16	4 or 5 or 6 or 7
17	3 and 12
18	13 or 14 or 15
19	16 or 17 or 18
20	exp infection/
21	wound dehiscence.mp. or exp wound dehiscence/
22	exp postoperative complication/ or wound breakdown.mp.
23	wound infection.mp. or exp wound infection/
24	surgical wound infection.mp. or exp surgical infection/
25	20 or 21 or 22 or 23 or 24
26	risk factor.mp. or exp risk factor/
27	exp prognosis/ or prognostic factor.mp.
28	26 or 27
29	19 and 25 and 28
30	limit 29 to "remove medline records"

Save Remove Combine with: AND OR

CINAHL searched 18th April 2024 (then updated to December 2024)

S32	S21 AND S27 AND S31	
S31	S28 OR S29 OR S30	**Expanders** - Apply equivalent subjects<br>**Search modes** - Boolean/Phrase
S30	(MH "Prognosis+")	**Expanders** - Apply equivalent subjects<br>**Search modes** - Boolean/Phrase
S29	"prognostic factor"	**Expanders** - Apply equivalent subjects<br>**Search modes** - Boolean/Phrase
S28	(MH "Risk Factors+") OR "risk factor"	**Expanders** - Apply equivalent subjects<br>**Search modes** - Boolean/Phrase
S27	S22 OR S23 OR S24 OR S25 OR S26	**Expanders** - Apply equivalent subjects<br>**Search modes** - Boolean/Phrase
S26	(MH "Infection+")	**Expanders** - Apply equivalent subjects<br>**Search modes** - Boolean/Phrase
S25	"wound breakdown"	**Expanders** - Apply equivalent subjects<br>**Search modes** - Boolean/Phrase
S24	(MH "Postoperative Complications+")	**Expanders** - Apply equivalent subjects<br>**Search modes** - Boolean/Phrase
S23	(MH "Surgical Wound Dehiscence") OR "wound dehiscence"	**Expanders** - Apply equivalent subjects<br>**Search modes** - Boolean/Phrase
S22	(MH "Wound Infection+") OR "wound infection"	**Expanders** - Apply equivalent subjects<br>**Search modes** - Boolean/Phrase
S21	S14 OR S15 OR S16 OR S17 OR S18 OR S19 OR S20	**Expanders** - Apply equivalent subjects<br>**Search modes** - Boolean/Phrase
S20	TI (episiotomy or episiotomies) OR AB (episiotomy or episiotomies)	**Expanders** - Apply equivalent subjects<br>**Search modes** - Boolean/Phrase
S19	(MM "Episiotomy")	**Expanders** - Apply equivalent subjects<br>**Search modes** - Boolean/Phrase
S18	TI ((childbirth or birth or labo?r or deliver*) and related and (perineal or perineum) and (tear* or injur* or trauma or lacerat* or wound* or rupture*)) OR AB ((childbirth or birth or labo?r or deliver*) and related and (perineal or perineum) and (tear* or injur* or trauma or lacerat* or wound* or rupture*))	**Expanders** - Apply equivalent subjects<br>**Search modes** - Boolean/Phrase

S17	AB ((third or 3rd or 4th or fourth) and degree and (perineal or perineum) and (tear* or injur* or trauma or lacerat* or wound* or rupture*)) OR TI ((third or 3rd or 4th or fourth) and degree and (perineal or perineum) and (tear* or injur* or trauma or lacerat* or wound* or rupture*))	**Expanders** - Apply equivalent subjects<br>**Search modes** - Boolean/Phrase
S16	TI (OASIS or OASI) OR AB (OASIS OR OASI)	**Expanders** - Apply equivalent subjects<br>**Search modes** - Boolean/Phrase
S15	TI (obstetric* and anal and sphincter and (tear* or injur* or trauma or lacerat* or wound* or rupture*)) OR AB (obstetric* and anal and sphincter and (tear* or injur* or trauma or lacerat* or wound* or rupture*))	**Expanders** - Apply equivalent subjects<br>**Search modes** - Boolean/Phrase
S14	S8 AND S13	**Expanders** - Apply equivalent subjects<br>**Search modes** - Boolean/Phrase
S13	S9 OR S10 OR S11 OR S12	**Expanders** - Apply equivalent subjects<br>**Search modes** - Boolean/Phrase
S12	TI (birth or childbirth or labo#r) OR AB (birth or childbirth or labo#r)	**Expanders** - Apply equivalent subjects<br>**Search modes** - Boolean/Phrase
S11	(MH "Childbirth"+)	**Expanders** - Apply equivalent subjects<br>**Search modes** - Boolean/Phrase
S10	(MH "Delivery, Obstetric+")	**Expanders** - Apply equivalent subjects<br>**Search modes** - Boolean/Phrase
S9	(MH "Labor Complications+")	**Expanders** - Apply equivalent subjects<br>**Search modes** - Boolean/Phrase
S8	S1 OR S2 OR S7	**Expanders** - Apply equivalent subjects<br>**Search modes** - Boolean/Phrase
S7	S5 AND S6	**Expanders** - Apply equivalent subjects<br>**Search modes** - Boolean/Phrase
S6	S3 OR S4	**Expanders** - Apply equivalent subjects<br>**Search modes** - Boolean/Phrase
S5	(MH "Wounds and Injuries+")	**Expanders** - Apply equivalent subjects<br>**Search modes** - Boolean/Phrase
S4	(MM "Anus")	**Expanders** - Apply equivalent subjects<br>**Search modes** - Boolean/Phrase
S3	(MM "Perineum")	**Expanders** - Apply equivalent subjects<br>**Search modes** - Boolean/Phrase

S2	(MH "Anus/IN")	**Expanders** - Apply equivalent subjects<br>**Search modes** - Boolean/Phrase
S1	(MH "Perineum/IN") OR TI((perineal or perineum) and ((tear* or injur* or trauma or lacerat* or wound* or rupture*)) OR AB ((perineal or perineum) and (tear* or injur* or trauma or lacerat* or wound* or rupture*))	

Web of Science Core Collection searched 18th April 2024 (then updated to December 2024)

27
#24 AND #25 AND #26
26
(((ALL=(risk factor*)) OR ALL=(risk-factor*)) OR TS=(prognosis)) OR TS=(prognostic factor)
25
(((((TS=(infection)) OR TS=(wound dehiscence)) OR TS=(wound breakdown)) OR TS=(wound complication)) OR TS=(postoperative complication*)) OR TS=(wound infection)
24
#4 or #5 or #6 or #7 or #10 or #11 or #12 or #13 or #14 or #23
23
#9 and #22
22
#17 or #19 or #20 or #21
21
(TI=((vagina or vaginal) (wound* or tear* or rupture* or fissure* or lacerat* or injur* or damage* or trauma*))) OR AB=((vagina or vaginal) (wound* or tear* or rupture* or fissure* or lacerat* or injur* or damage* or trauma*))
20
(TI=((vulva or vulvar or labial) (wound* or tear* or rupture* or fissure* or lacerat* or injur* or damage* or trauma*))) OR AB=((vagina or vaginal) (wound* or tear* or rupture* or fissure* or lacerat* or injur* or damage* or trauma*))
19
#15 and #18
18
TS=wounds
17
#15 AND #16
16
TS=injuries
15
TS=vulva
14
(TI=(episiotom*)) OR AB=(episiotom*)
13

TS=(episiotomy)

12

(TS=(OASI)) OR TS=(OASIS)

11

(TI=((obstetric* and anal and sphincter and (tear* or injur* or trauma* or damage* or wound* or rupture* of fissure* or lacerat*)))) OR AB=((obstetric* and anal and sphincter and (tear* or injur* or trauma* or damage* or wound* or rupture* of fissure* or lacerat*)))

10

#8 AND #9

9

TS=(obstetric labour complications)

8

(TS=(anal canal)) AND TS=(injuries)

7

TI=((perineum or perineal) (trauma*or injur* or wound* or lacerat* or fissure* or rupture* or damage* or tear*)) OR AB=((perineum or perineal) (trauma*or injur* or wound* or lacerat* or fissure* or rupture* or damage* or tear*))

6

TS=(perineal trauma)

5

#1 AND #3

4

#1 AND #2

3

TS= lacerations

2

TS=("wounds and injuries")

1

(TS=(perineum))
