# Supplementary figures and images for: Prognostic factors for wound complications after childbirth‐related perineal trauma: A systematic review and meta‐analysis
Source: Acta Obstet Gynecol Scand. 2025 Aug 20;105(7):1247–64. doi: 10.1111/aogs.70041 (PMC13308966; doi:10.1111/aogs.70041)

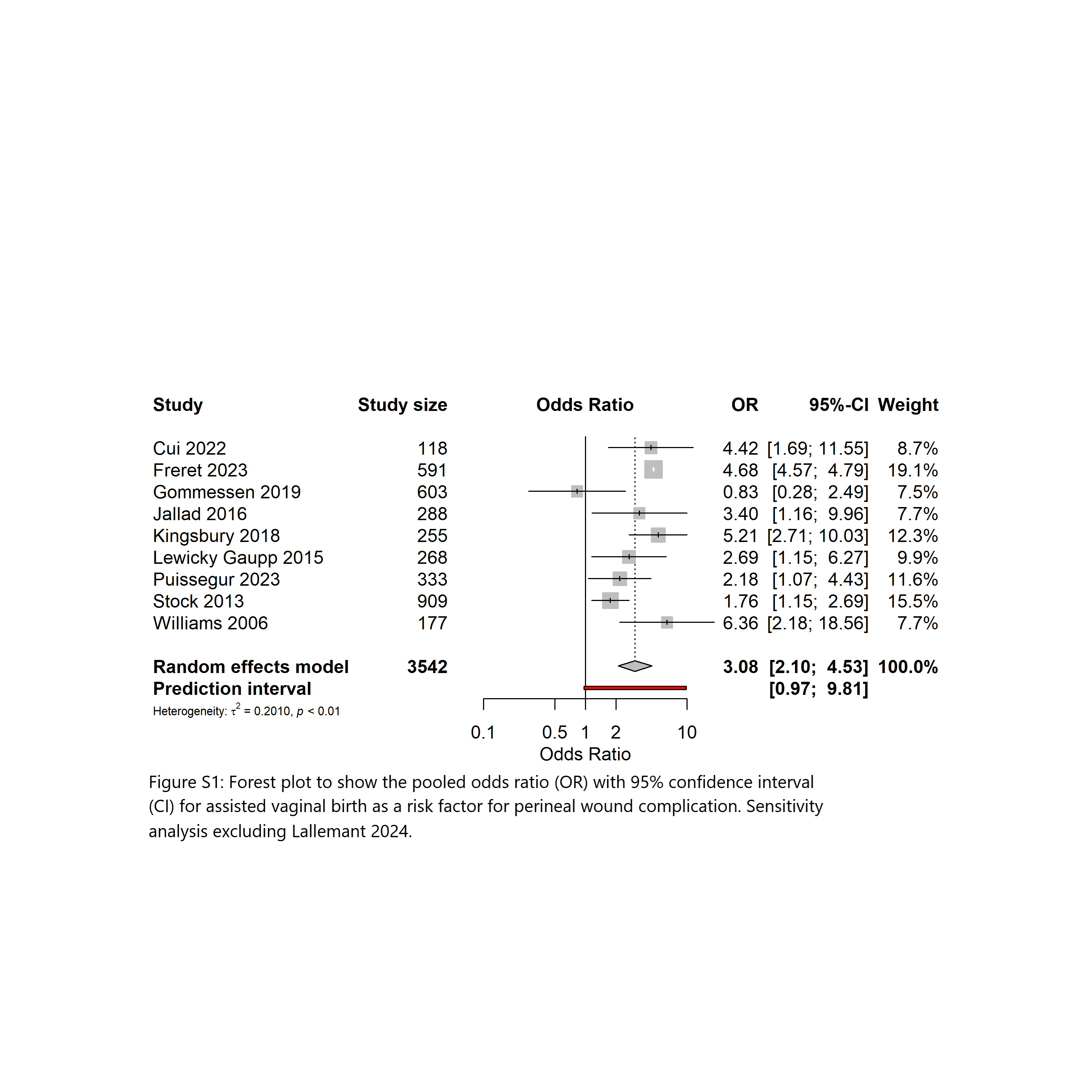

Supplement: Supplementary file 2 — Figure S1. Forest plot to show the pooled odds ratio (OR) with 95% confidence interval (CI) for assisted vaginal birth as a risk factor for perineal wound complication. Sensitivity analysis excluding Lallemant 2024. [file AOGS-105-1247-s004.tiff]

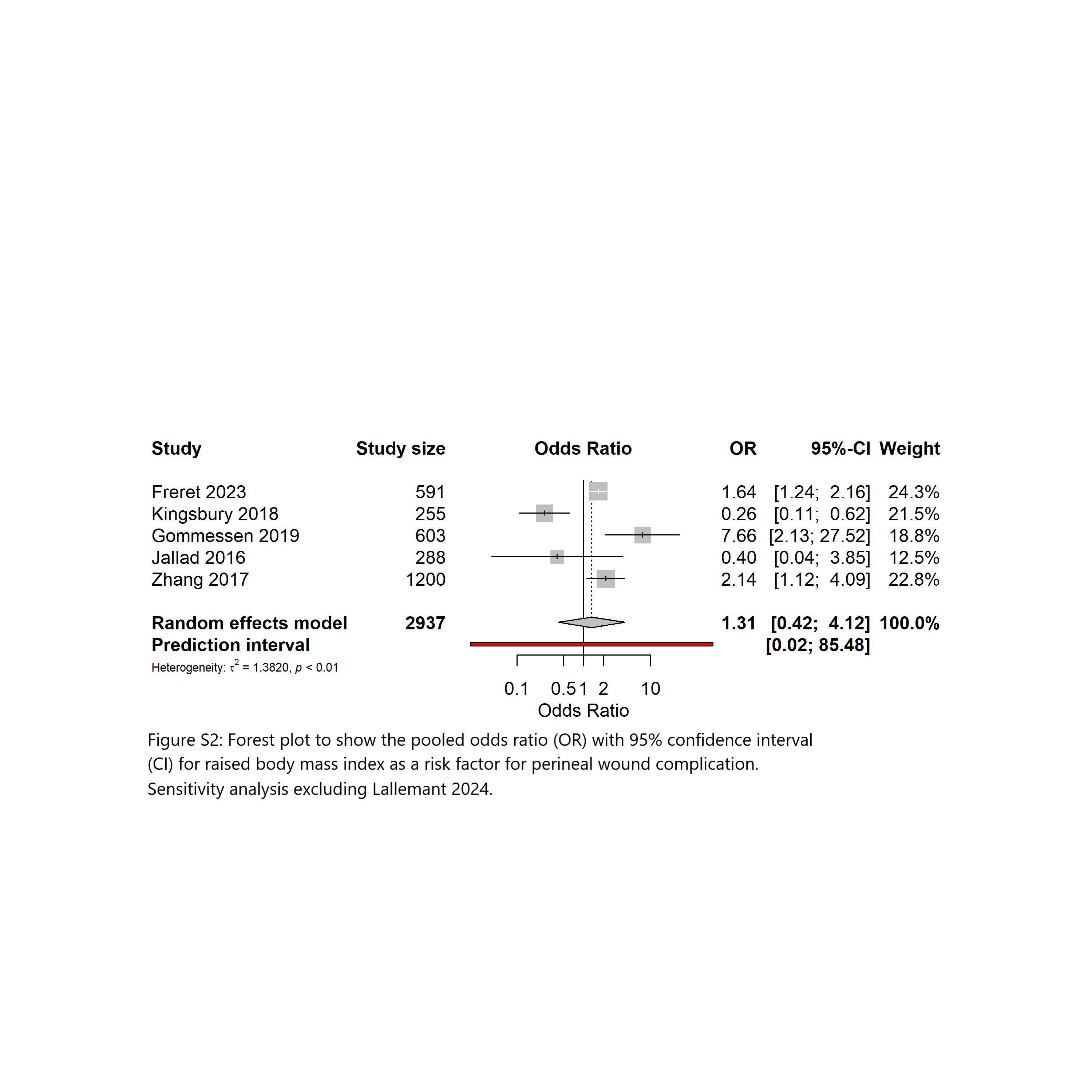

Supplement: Supplementary file 3 — Figure S2. Forest plot to show the pooled odds ratio (OR) with 95% confidence interval (CI) for raised body mass index as a risk factor for perineal wound complication. Sensitivity analysis excluding Lallemant 2024. [file AOGS-105-1247-s006.tiff]

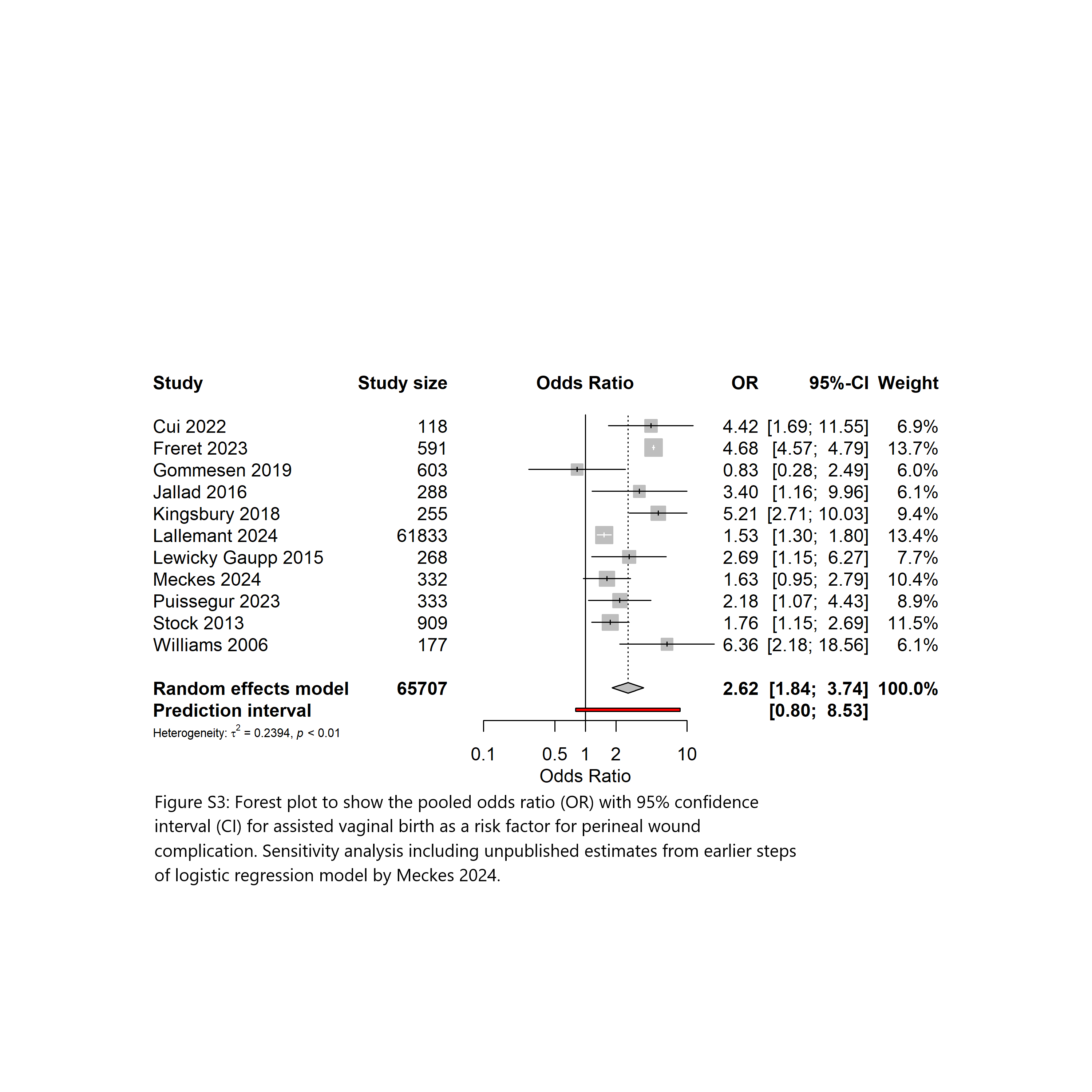

Supplement: Supplementary file 4 — Figure S3. Forest plot to show the pooled odds ratio (OR) with 95% confidence interval (CI) for assisted vaginal birth as a risk factor for perineal wound complication. Sensitivity analysis including unpublished estimates from earlier steps of logistic regression model by Meckes 2024. [file AOGS-105-1247-s003.tiff]

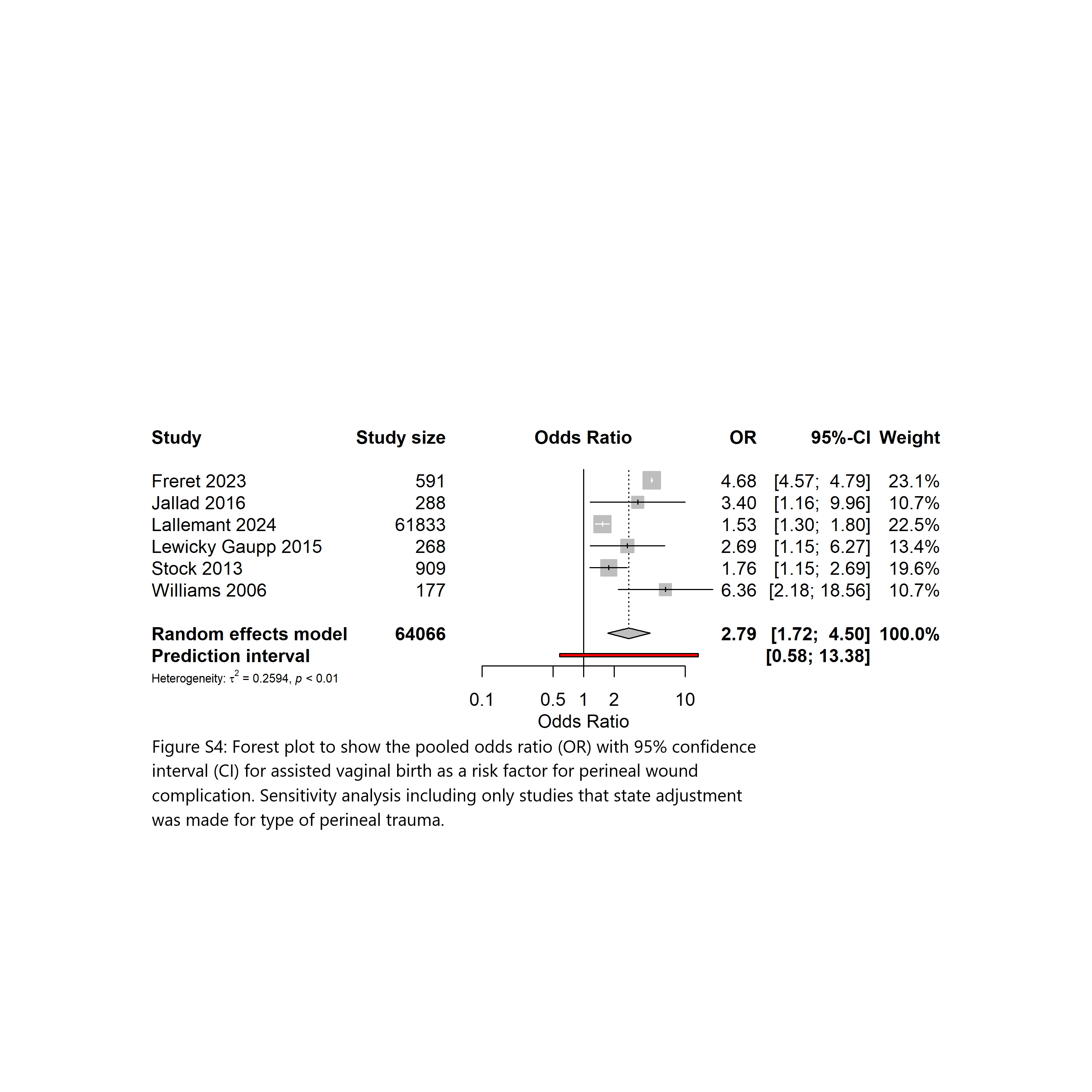

Supplement: Supplementary file 5 — Figure S4. Forest plot to show the pooled odds ratio (OR) with 95% confidence interval (CI) for assisted vaginal birth as a risk factor for perineal wound complication. Sensitivity analysis including only studies that state adjustment was made for type of perineal trauma. [file AOGS-105-1247-s007.tiff]

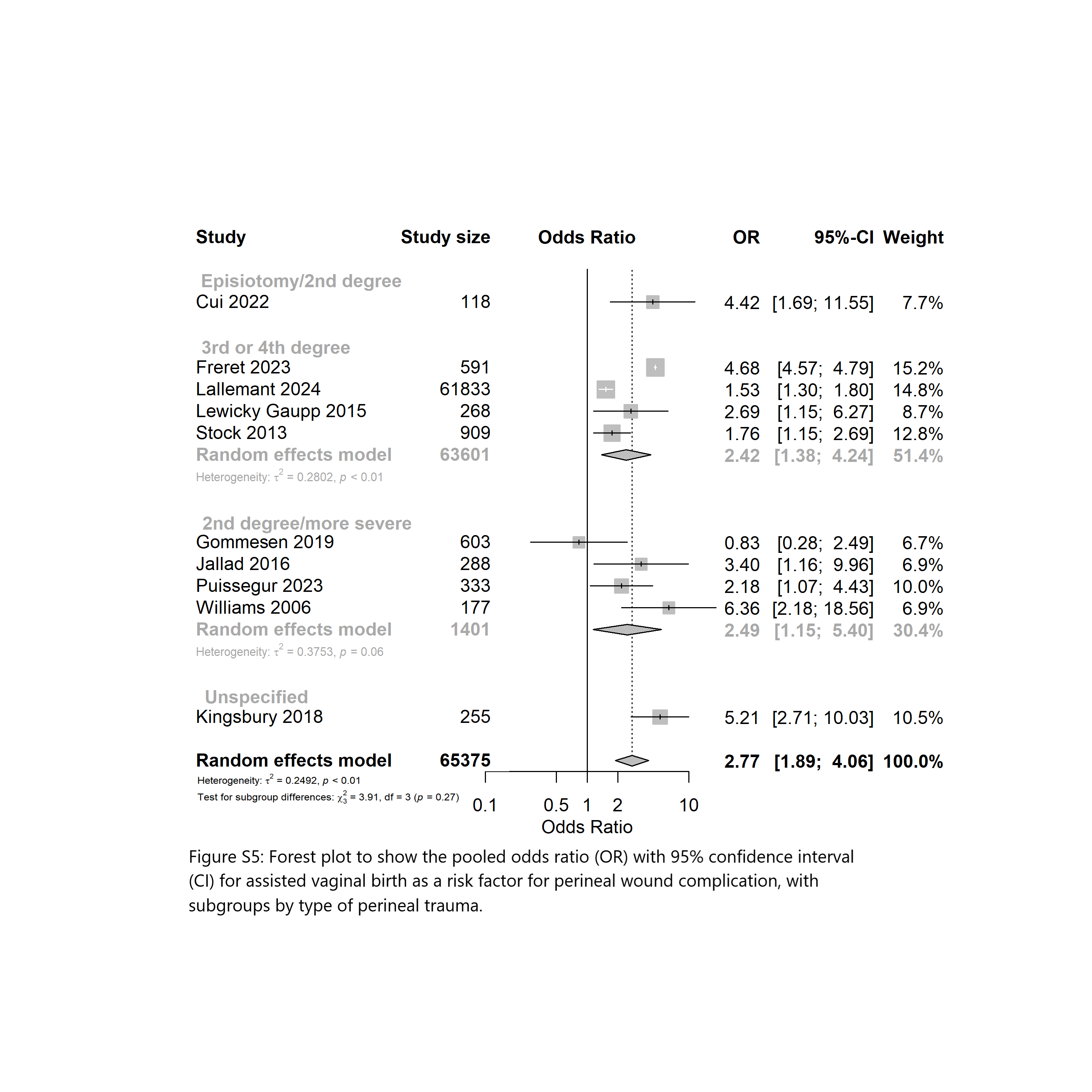

Supplement: Supplementary file 6 — Figure S5. Forest plot to show the pooled odds ratio (OR) with 95% confidence interval (CI) for assisted vaginal birth as a risk factor for perineal wound complication, with subgroups by type of perineal trauma. [file AOGS-105-1247-s005.tiff]
