# Supplementary material for: Prognostic factors for wound complications after childbirth‐related perineal trauma: A systematic review and meta‐analysis
Source: Acta Obstet Gynecol Scand. 2025 Aug 20;105(7):1247–64. doi: 10.1111/aogs.70041 (PMC13308966; doi:10.1111/aogs.70041)
Supplement: Supplementary file 7 — Table S1. Risk of bias assessments for included studies. [file AOGS-105-1247-s001.docx]

| **Study** | **Study participation** | **Study attrition** | **Prognostic factor measurement** | **Outcome measurement** | **Adjustment for other prognostic factors** | **Statistical analysis and reporting** | **Total number of 'low' judgments** |
| --- | --- | --- | --- | --- | --- | --- | --- |
| **Cui 2022** | Moderate | Low | Moderate | Moderate | Low | Low | 3 |
| **Freret 2023** | Low | Moderate | Low | Moderate | Low | Low | 4 |
| **Gommesen 2019** | Low | Moderate | Low | Low | Low | Low | 5 |
| **Jallad 2016** | Low | Low | Low | Moderate | Low | Low | 5 |
| **Kingsbury 2018** | Moderate | Low | Moderate | Low | Low | Low | 4 |
| **Lallemant 2024** | Low | Moderate | Moderate | Moderate | Low | Low | 3 |
| **Lewicky-Gaupp 2015** | Low | Moderate | Low | Low | Low | High | 4 |
| **Meckes 2024** | Low | Low | Moderate | Moderate | Low | Low | 4 |
| **Propst 2023** | Low | Low | Low | Moderate | Low | Moderate | 4 |
| **Puissegur 2023** | Moderate | Low | Moderate | Moderate | Low | Low | 3 |
| **Stock 2012** | Low | Moderate | Low | Moderate | Low | Low | 4 |
| **Thongtip 2023** | Low | Low | Moderate | Low | Low | Low | 5 |
| **Wilkie 2018** | Low | Low | Low | Moderate | Low | High | 4 |
| **Williams 2006** | Moderate | Low | Low | Moderate | Low | Low | 4 |
| **Zhang 2017** | Moderate | Low | High | High | Low | High | 2 |
